# Machine learning analysis with population data for the associations of preterm birth with temporomandibular disorder and gastrointestinal diseases

**DOI:** 10.1371/journal.pone.0296329

**Published:** 2024-01-02

**Authors:** Kwang-Sig Lee, In-Seok Song, Eun Sun Kim, Jisu Kim, Sohee Jung, Sunwoo Nam, Ki Hoon Ahn

**Affiliations:** 1 AI Center, Korea University College of Medicine, Anam Hospital, Seoul, Korea; 2 Department of Oral and Maxillofacial Surgery, Korea University Anam Hospital, Seoul, Korea; 3 Department of Gastroenterology, Korea University College of Medicine, Anam Hospital, Seoul, Korea; 4 Department of Statistics, Korea University College of Political Science and Economics, Seoul, Korea; 5 Department of Obstetrics and Gynecology, Korea University College of Medicine, Anam Hospital, Seoul, Korea; University of Catanzaro, ITALY

## Abstract

This study employs machine learning analysis with population data for the associations of preterm birth (PTB) with temporomandibular disorder (TMD) and gastrointestinal diseases. The source of the population-based retrospective cohort was Korea National Health Insurance claims for 489,893 primiparous women with delivery at the age of 25–40 in 2017. The dependent variable was PTB in 2017. Twenty-one predictors were included, i.e., demographic, socioeconomic, disease and medication information during 2002–2016. Random forest variable importance was derived for finding important predictors of PTB and evaluating its associations with the predictors including TMD and gastroesophageal reflux disease (GERD). Shapley Additive Explanation (SHAP) values were calculated to analyze the directions of these associations. The random forest with oversampling registered a much higher area under the receiver-operating-characteristic curve compared to logistic regression with oversampling, i.e., 79.3% vs. 53.1%. According to random forest variable importance values and rankings, PTB has strong associations with low socioeconomic status, GERD, age, infertility, irritable bowel syndrome, diabetes, TMD, salivary gland disease, hypertension, tricyclic antidepressant and benzodiazepine. In terms of max SHAP values, these associations were positive, e.g., low socioeconomic status (0.29), age (0.21), GERD (0.27) and TMD (0.23). The inclusion of low socioeconomic status, age, GERD or TMD into the random forest will increase the probability of PTB by 0.29, 0.21, 0.27 or 0.23. A cutting-edge approach of explainable artificial intelligence highlights the strong associations of preterm birth with temporomandibular disorder, gastrointestinal diseases and antidepressant medication. Close surveillance is needed for pregnant women regarding these multiple risks at the same time.

## Introduction

Preterm birth (PTB) is defined as “birth before 37 weeks of gestation” [1] (abbreviations listed in S1 Table). It consists of PTB with premature rupture of membranes (PROM), preterm labor and birth without PROM and other PTB. Its frequency varies from 5% to 9% in developed countries but in general it has increased with the growth of indicated PTB and due to other factors, e.g., 9.5% in 1981 to 12.7% in 2005 for the United States [1]. Several studies reported that preterm has positive associations with maternal depression and stress [2–5]. Based on a review, preterm birth was a major risk factor for maternal depression, neurodevelopmental delay and childhood disability [2]. According to an animal experiment, prenatal maternal stress was a major cause of preterm birth and neonatal immunity in mice [3]. The results of the studies above agree with those of two other reviews stating that maternal depression and stress during pregnancy affect preterm birth [4, 5].

Likewise, temporomandibular disorder (TMD) and gastrointestinal disease are expected to have positive associations with depression and stress [6–11]. TMD can be defined as “disorder affecting joints and muscles” [6]. Its symptoms are joint pain, muscle pain and limited mouth opening, whereas its etiological causes are occlusion, trauma, pain stimulus, parafunctional activity and psychological stress [6]. A prospective study of female students before the university entrance reported a positive relationship between stress and TMD [7]. This positive relationship between stress and TMD was affirmed by a review of 33 original studies [8] and a survey of 112 participants in a general hospital [9]. Also, two other reviews highlighted a positive association between depression and gastrointestinal disease such as gastroesophageal reflux disease (GERD) and irritable bowel syndrome [10, 11]. Based on the findings above, one would expect a positive relationship among PTB, TMD and GERD. But no literature has been available on this topic. In this context, this study employs machine learning analysis with population data for the associations of PTB with TMD and gastrointestinal diseases.

## Methods

### Participants and variables

The source of the population-based retrospective cohort was Korea National Health Insurance claims for 489,893 primiparous women with delivery at the age of 25–40 in 2017. This retrospective cohort study was approved by the Institutional Review Board (IRB) of Korea University Anam Hospital on June 12, 2023 (2020AN0014). Informed consent was waived by the IRB. The data were accessed for the research during July 1, 2023-August 31, 2023. The authors did not have access to information that could identify individual participants during or after data collection. The dependent variables were four categories of PTB (birth before 37 weeks of gestation) in 2017 based on the ICD-10 Code: PTB 1—PTB with PROM only; PTB 2—preterm labor and birth without PROM; PTB 3—PTB 1 or PTB 2; PTB 4—PTB 3 or other indicated PTB (S2 Table). The 21 independent variables were: (1) two demographic/socioeconomic predictors in 2016, i.e., age, low socioeconomic status with the range of 1 (the highest) to 20 (the lowest) in terms of an insurance fee; (2) five dental diseases for any of the years 2002–2016, including dental cavity, periodontitis, salivary gland disease, tooth loss, TMD; (3) four gastrointestinal diseases for any of the years 2002–2016, i.e., Crohn’s disease, GERD, irritable bowel syndrome, ulcerative colitis; (4) four obstetric conditions for any of the years 2002–2016, that is, infertility, hypertension, diabetes, gestational diabetes; (5) six medication predictors in 2016, i.e., benzodiazepine, calcium channel blocker, nitrate, progesterone, sleeping pill, tricyclic antidepressant. These 21 independent variables were selected according to previous studies and data availability. The disease and medication data were screened from ICD-10 and ATC codes, respectively (S2 and S3 Tables).

### Machine learning analysis

Logistic regression and the random forest were used for the prediction of PTB. A random forest is a group of decision trees which make majority votes on the dependent variable (“bootstrap aggregation”) [12, 13]. The 489,893 cases with full information were divided into training and validation sets with an 80:20 ratio (391,914 vs. 97,979 cases). The validation criteria were accuracy (a ratio of correct predictions among 97,979 cases) and the area under the receiver-operating-characteristic curve (AUC) (area under the plot of sensitivity vs. 1—specificity). Random forest variable importance was derived for finding important predictors of PTB and evaluating the strengths of its associations with the predictors. Random forest Shapley Additive Explanation (SHAP) values were calculated to analyze the directions of these associations. The permutation importance of a predictor indicates the decrease of model accuracy from data permutation on the predictor. It is an average over all trees with a value of 0 to 1 in the case of the random forest. The SHAP value of a predictor for a participant measures the difference between what machine learning predicts for the probability of PTB with and without the predictor. For example, let us assume that the SHAP values of TMD for PTB have the range of (−0.10, 0.23). Here, some participants have SHAP values as low as −0.10, and other participants have SHAP values as high as 0.23. The inclusion of a predictor (TMD) into machine learning will decrease or increase the probability of the dependent variable (PTB) by the range of −0.10 and 0.23. In other words, there exists a positive association between TMD and PTB in general [12].

In practice, experts in artificial intelligence use random forest permutation importance to derive the rankings and values of all predictors for the prediction of the dependent variable. Then, they employ the SHAP plots to evaluate the directions of associations between the predictors and the dependent variable. Linear or logistic regression used to play this role before the SHAP approach took it over. This is because the SHAP approach has a notable strength compared to linear or logistic regression: the former considers all realistic scenarios, unlike the latter with an unrealistic assumption of *ceteris paribus*, i.e., “all the other variables staying constant”. Let us assume that there are three predictors of PTB, i.e., low socioeconomic status, GERD and TMD. As defined above, the SHAP value of TMD for PTB for a particular participant is the difference between what machine learning predicts for the probability of PTB with and without TMD for the participant. Here, the SHAP value for the participant is the average of the following four scenarios for the participant: (1) low socioeconomic status excluded, GERD excluded; (2) low socioeconomic status included, GERD excluded; (3) low socioeconomic status excluded, GERD included; and (4) low socioeconomic status included, GERD included (13). Finally, it can be noted that R-Studio 1.3.959 (R-Studio Inc.: Boston, United States) was employed for the analysis during January 1, 2023-February 28, 2023.

## Results

Descriptive statistics are shown in Table 1. The corresponding means and standard deviations of age and socioeconomic status in 2016 were 31.10 and 3.59. The respective proportions of those with GERD, TMD and tricyclic antidepressants during 2002–2016 were 42.1% (207,804), 3.9% (19,345) and 10.3% (50,305). Model performance is presented in Table 2. The random forest with oversampling registered much higher AUC compared to logistic regression with oversampling, i.e., 80.4% vs. 51.6% (PTB1), 86.2% vs. 57.2% (PTB2), 79.3% vs. 52.7% (PTB3), 79.3% vs. 53.1% (PTB4). Based on random forest variable importance values and rankings in Table 3, PTB1, PTB2, PTB3 and PTB4 have strong associations with low socioeconomic status, GERD, age, infertility, irritable bowel syndrome, diabetes, TMD, salivary gland disease, hypertension, tricyclic antidepressant and benzodiazepine. In terms of max SHAP values for PTB1, PTB2, PTB3 and PTB4 in Table 4, these associations were positive, e.g., low socioeconomic status (0.29), age (0.21), GERD (0.27) and TMD (0.23) for PTB4 in Table 4. The inclusion of low socioeconomic status, age, GERD or TMD into the random forest will increase the probability of PTB4 by 0.29, 0.21, 0.27 or 0.23.

**Table 1:** Descriptive statistics on categorical variables.

Total 489893	*No*	*Yes*	*Yes (%)*
Benzodiazepine	288746	201147	41.1
Calcium Channel Blocker	488567	1326	0.3
Crohn’s Disease	487166	2727	0.6
Dental Cavity	484214	5679	1.2
Diabetes	460368	29525	6.0
Gastroesophageal Reflux Disease	282089	207804	42.1
Gestational Diabetes	468366	21527	4.4
Hypertension	476350	13543	2.7
Infertility	417272	72621	14.7
Irritable Bowel Syndrome	436207	53686	10.9
Nitrate	488875	1018	0.2
Periodontitis	484913	4980	1.0
Progesterone	421471	68422	14.0
Salivary Gland Disease	472881	17012	3.4
Sleeping Pill	465310	24583	5.0
Temporomandibular Disorder	470548	19345	3.9
Tooth Loss	489581	312	0.1
Tricyclic Antidepressant	439588	50305	10.3
Ulcerative Colitis	487484	2409	0.5

**Table 2:** Model performance.

	*PTB1*	*PTB2*	*PTB3*	*PTB4*
Logistic Regression				
Accuracy	0.5616	0.6051	0.5691	0.5726
Area Under the Curve	0.5157	0.5721	0.5270	0.5313
Random Forest				
Accuracy	0.8130	0.8674	0.8040	0.8036
Area Under the Curve	0.8035	0.8617	0.7934	0.7928

**Table 3:** Random forest variable importance.

	PTB1		PTB2		PTB3		PTB4	
	*Rank*	*VI*	*Rank*	*VI*	*Rank*	*VI*	*Rank*	*VI*
Age	3	0.2072	3	0.1657	3	0.1950	3	0.1895
Benzodiazepine	12	0.0109	7	0.0291	12	0.0123	11	0.0123
Calcium Channel Blocker	19	0.0019	20	0.0018	19	0.0020	20	0.0019
Crohn’s Disease	17	0.0061	17	0.0052	17	0.0062	17	0.0064
Dental Cavity	13	0.0108	14	0.0100	13	0.0109	13	0.0111
Diabetes	6	0.0366	6	0.0387	6	0.0394	6	0.0394
Gastroesophageal Reflux Disease	2	0.2239	2	0.2360	2	0.2266	2	0.2279
Gestational Diabetes	16	0.0068	15	0.0095	16	0.0079	16	0.0078
Hypertension	9	0.0207	10	0.0206	9	0.0206	9	0.0196
Infertility	4	0.0533	4	0.0827	4	0.0601	4	0.0612
Irritable Bowel Syndrome	5	0.0508	5	0.0549	5	0.0521	5	0.0551
Nitrate	20	0.0017	19	0.0018	20	0.0019	19	0.0020
Periodontitis	15	0.0094	16	0.0085	14	0.0101	14	0.0102
Progesterone	11	0.0142	12	0.0133	11	0.0141	12	0.0116
Salivary Gland Disease	8	0.0300	9	0.0217	8	0.0308	8	0.0314
Sleeping Pill	14	0.0103	13	0.0107	15	0.0099	15	0.0102
Socioeconomic Status	1	0.2479	1	0.2399	1	0.2419	1	0.2473
Temporomandibular Disorder	7	0.0333	8	0.0281	7	0.0337	7	0.0330
Tooth Loss	21	0.0008	21	0.0008	21	0.0008	21	0.0008
Tricyclic Antidepressant	10	0.0184	11	0.0164	10	0.0185	10	0.0160
Ulcerative Colitis	18	0.0050	18	0.0044	18	0.0051	18	0.0052

Note: Red Top-5, Blue Top-10, VI Variable Importance

**Table 4:** Random forest Shapley additive explanations (SHAP) values.

	PTB1		PTB2		PTB3		PTB4	
	*Max*	*Min*	*Max*	*Min*	*Max*	*Min*	*Max*	*Min*
Age	0.2403	-0.2565	0.2615	-0.3519	0.2355	-0.2342	0.2145	-0.2149
Benzodiazepine	0.2142	-0.1640	0.1493	-0.2552	0.2135	-0.1608	0.2165	-0.1517
Calcium Channel Blocker	0.1128	-0.1069	0.1120	-0.0680	0.1651	-0.1161	0.0793	-0.0814
Crohn’s Disease	0.3680	-0.1723	0.2814	-0.2017	0.3486	-0.1394	0.3176	-0.1637
Dental Cavity	0.3087	-0.2617	0.3473	-0.3901	0.2375	-0.2172	0.2856	-0.2164
Diabetes	0.2664	-0.2643	0.3584	-0.2551	0.2081	-0.2631	0.2317	-0.2713
Gastroesophageal Reflux Disease	0.3030	-0.2293	0.2815	-0.3261	0.3162	-0.2140	0.2650	-0.2194
Gestational Diabetes	0.1541	-0.3009	0.2231	-0.3094	0.2272	-0.2691	0.1768	-0.3193
Hypertension	0.3076	-0.2385	0.3186	-0.2549	0.2924	-0.2303	0.2776	-0.2537
Infertility	0.3946	-0.1637	0.3328	-0.2571	0.3656	-0.1808	0.3596	-0.1784
Irritable Bowel Syndrome	0.2738	-0.2655	0.4237	-0.3078	0.2816	-0.2480	0.3269	-0.2983
Nitrate	0.0925	-0.0844	0.3500	-0.0085	0.2233	-0.0720	0.1035	-0.1019
Periodontitis	0.2034	-0.2726	0.3247	-0.2429	0.2809	-0.2380	0.1651	-0.2427
Progesterone	0.2381	-0.2137	0.2340	-0.2897	0.2508	-0.2006	0.2301	-0.2010
Salivary Gland Disease	0.3383	-0.2441	0.3540	-0.3022	0.2856	-0.2156	0.2909	-0.2298
Sleeping Pill	0.2167	-0.1960	0.1906	-0.2389	0.2164	-0.1725	0.2032	-0.1612
Socioeconomic Status	0.2854	-0.2031	0.2703	-0.2899	0.3079	-0.2089	0.2903	-0.1980
Temporomandibular Disorder	0.2716	-0.2535	0.2993	-0.3340	0.2402	-0.2910	0.2331	-0.2490
Tooth Loss	0.0484	-0.1582	0.2336	-0.1023	0.0614	-0.1649	0.0422	-0.1105
Tricyclic Antidepressant	0.2310	-0.2258	0.1711	-0.3004	0.2160	-0.1810	0.2545	-0.2050
Ulcerative Colitis	0.2715	-0.1710	0.3317	-0.1453	0.1673	-0.1273	0.1843	-0.1241

**Fig 1:**
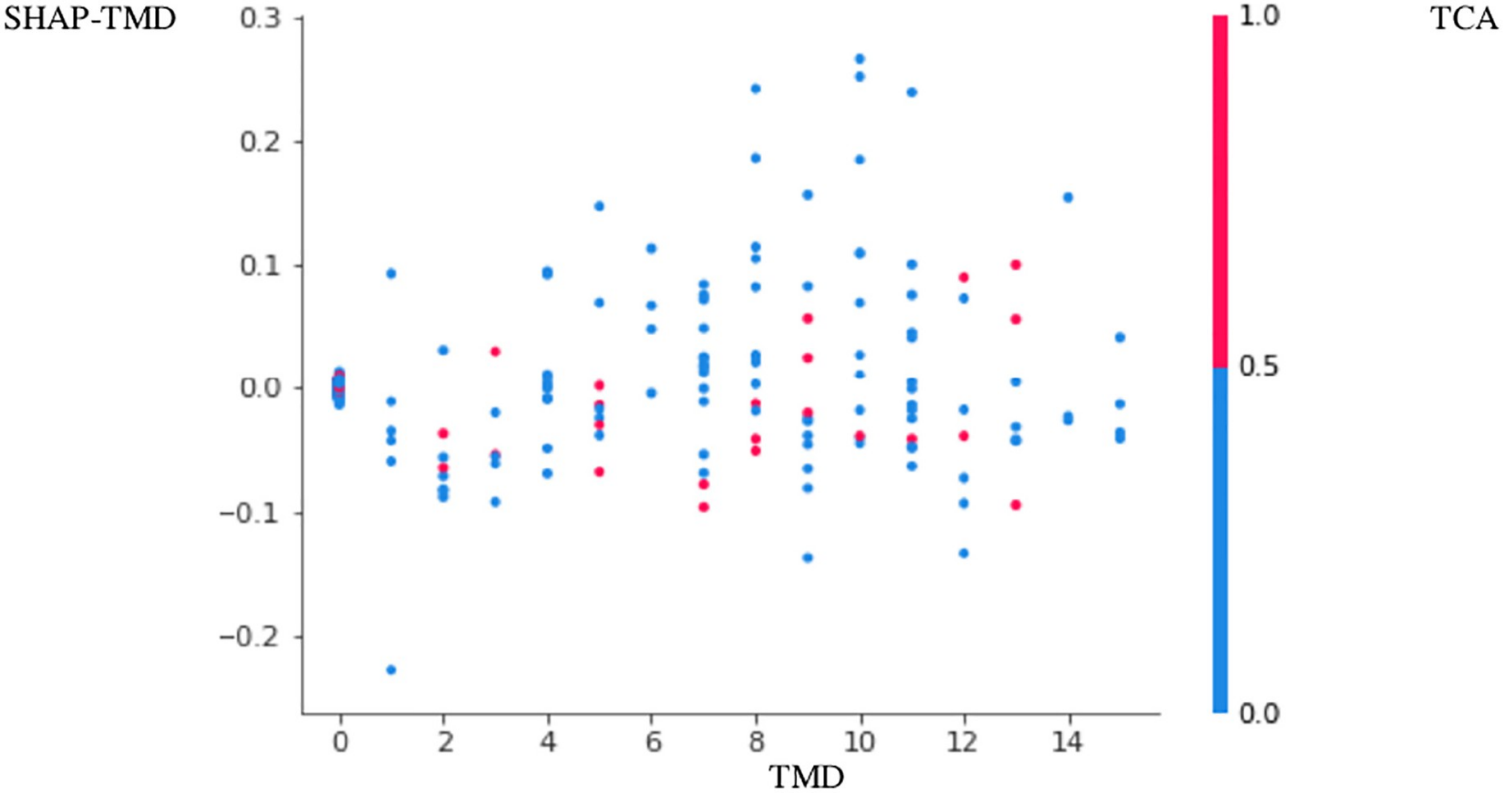
SHAP dependence plot PTB1–3,000 sampling. Legend: The positive association between PTB (preterm birth) and TMD (temporomandibular disease) was more apparent in Figs 1–4. Here, points with low TMD values and low SHAP values for PTB were positioned in the left bottom, while points with high TMD values and high SHAP values for PTB were positioned in the right top. These figures are called the SHAP dependence plots of TMD vs. PTB. In these figures, the blue (or red) color represents the absence (or presence) of TCA (tricyclic antidepressant) for a participant, which was found to have the highest correlation with TMD for the prediction of PTB. Here, points with low TMD values, the absence of TCA and low SHAP values for PTB were positioned in the left bottom, whereas points with high TMD values, the presence of TCA and high SHAP values for PTB were positioned in the right top.

**Fig 2:**
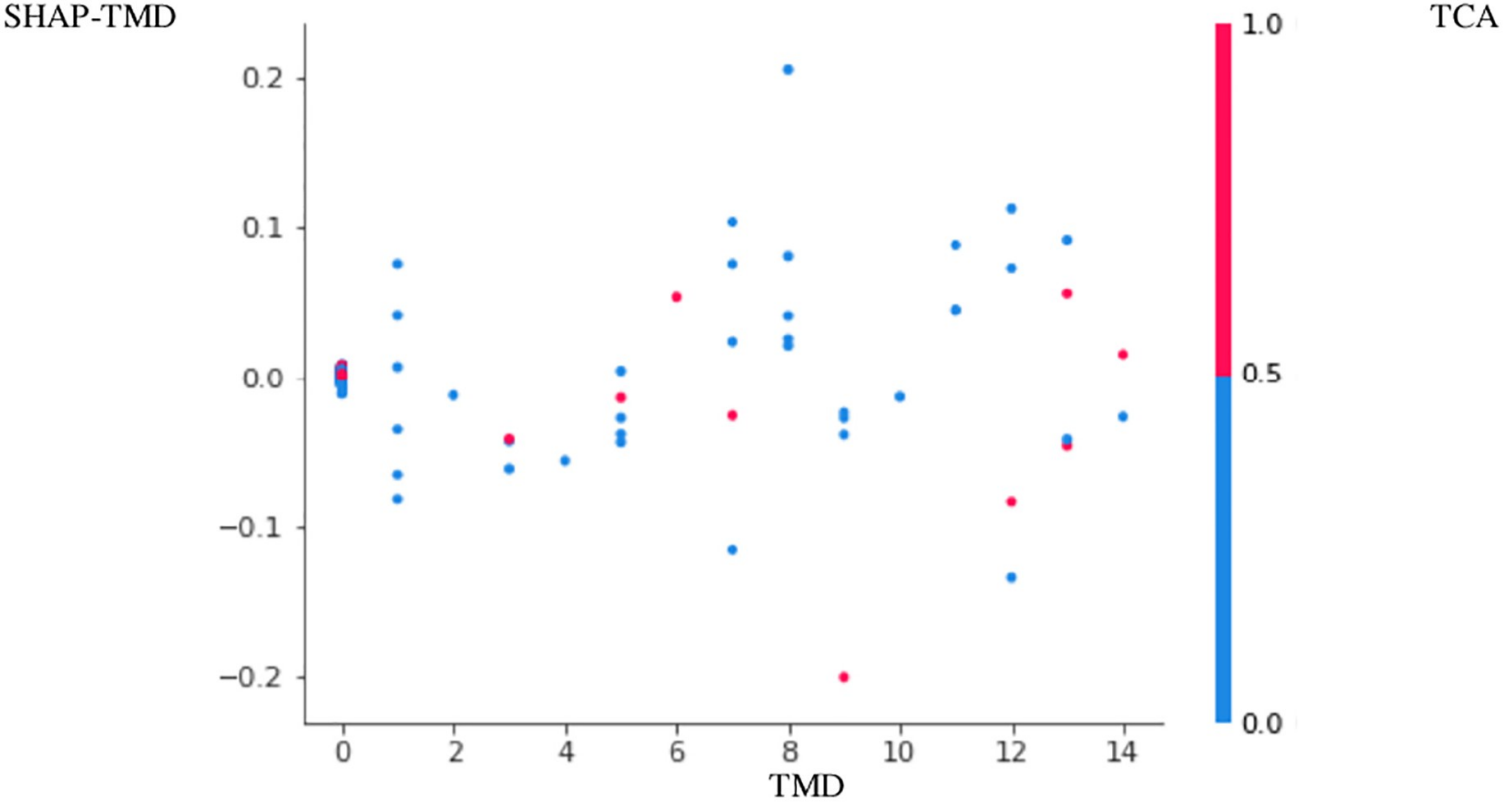
SHAP dependence plot PTB2–1,000 sampling.

**Fig 3:**
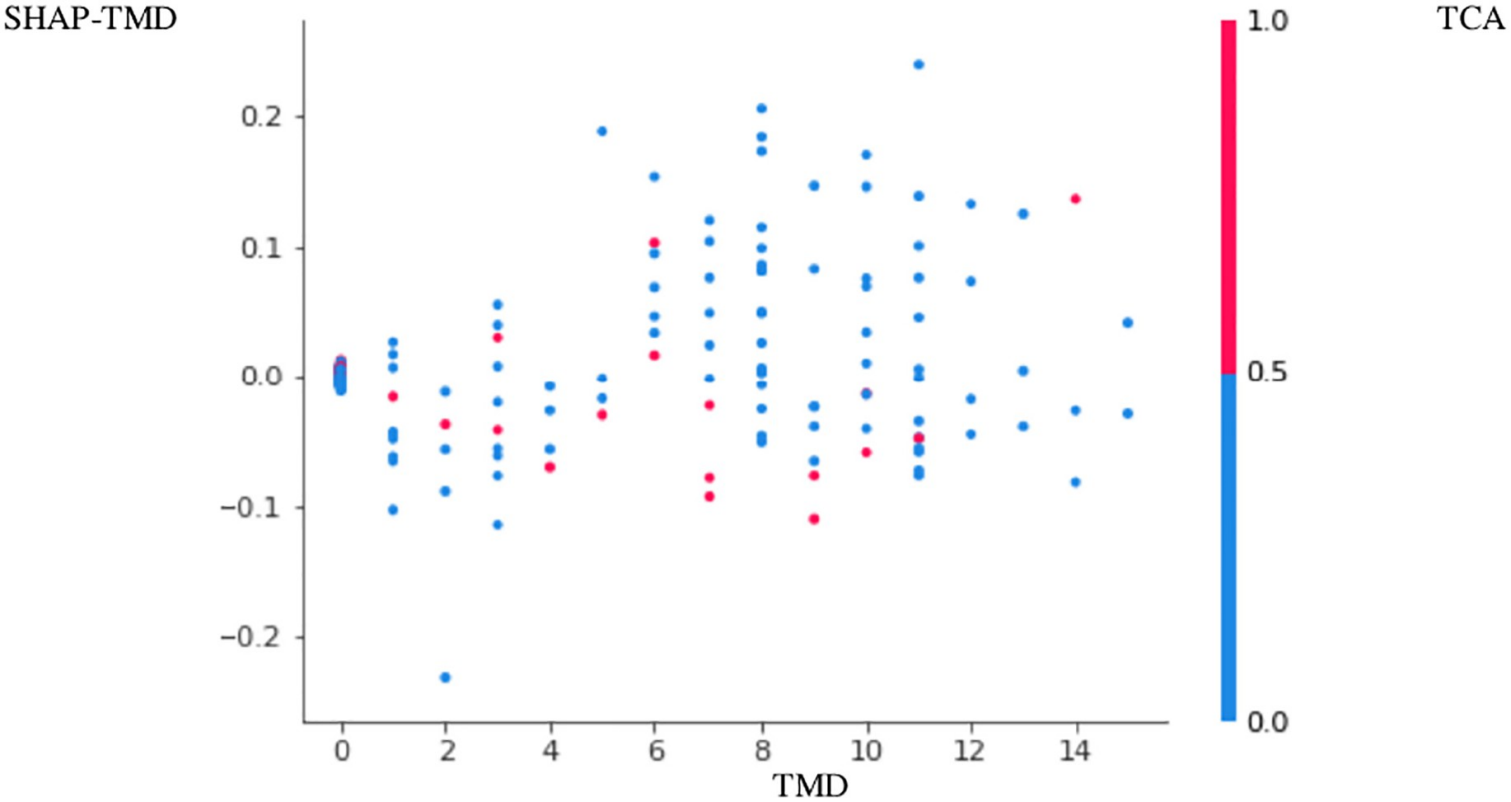
SHAP dependence plot PTB3–3,000 sampling.

**Fig 4:**
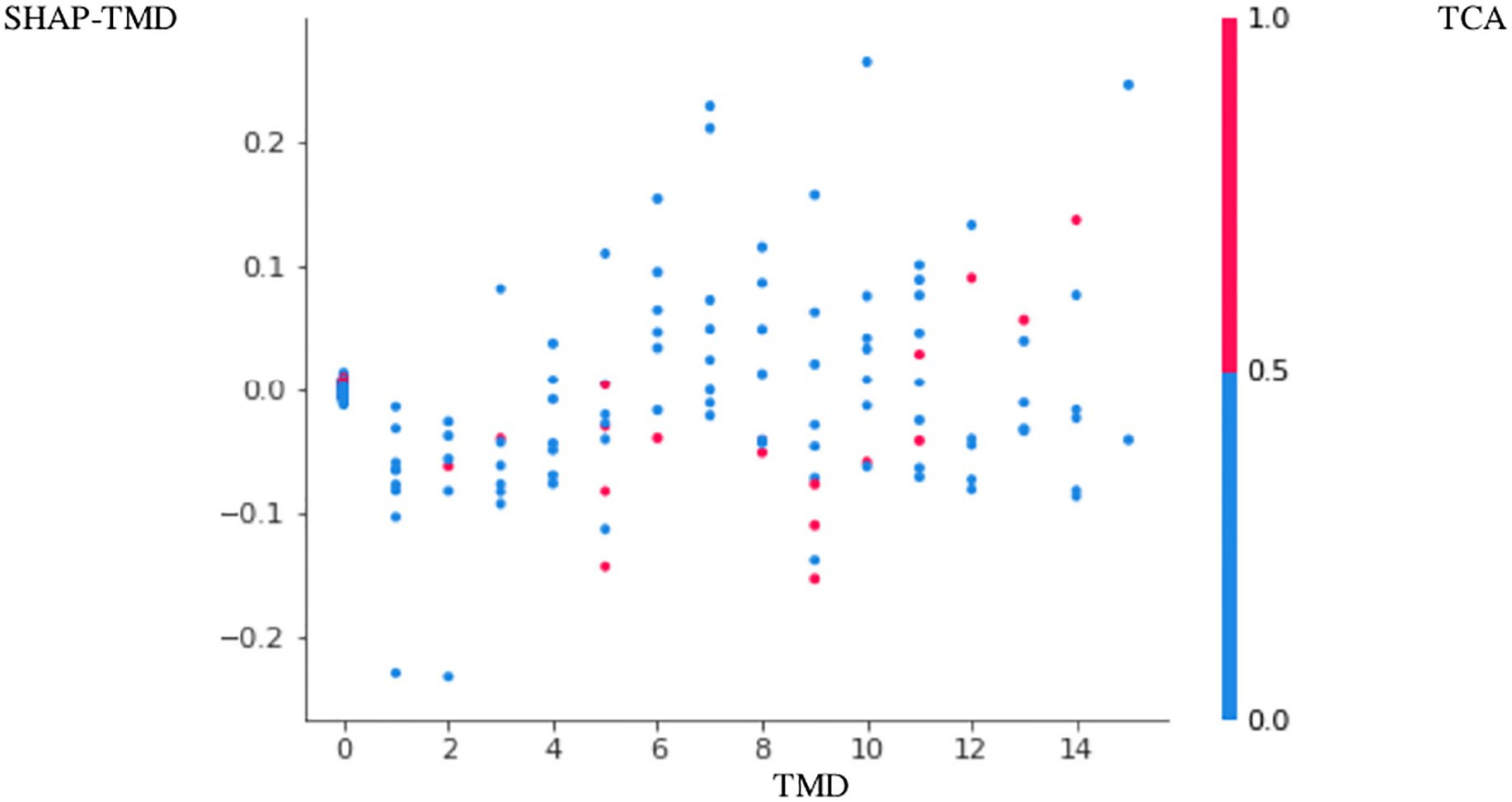
SHAP dependence plot PTB4–3,000 sampling.

The positive association between PTB and TMD was more apparent in Figs 1–4. Here, points with low TMD values and low SHAP values for PTB were positioned in the left bottom, while points with high TMD values and high SHAP values for PTB were positioned in the right top (Figs 1–4). These figures are called the SHAP dependence plots of TMD vs. PTB. In these figures, the blue (or red) color represents the absence (or presence) of tricyclic antidepressant for a participant, which was found to have the highest correlation with TMD for the prediction of PTB. Here, points with low TMD values, the absence of tricyclic antidepressant and low SHAP values for PTB were positioned in the left bottom, whereas points with high TMD values, the presence of tricyclic antidepressant and high SHAP values for PTB were positioned in the right top.

## Discussion

### Summary

Based on random forest variable importance values and rankings, PTB has strong associations with low socioeconomic status, GERD, age, infertility, irritable bowel syndrome, diabetes, TMD, salivary gland disease, hypertension, tricyclic antidepressant and benzodiazepine. Specifically, the positive association among PTB, TMD and tricyclic antidepressant was more apparent in the SHAP dependence plot: points with low TMD values, the absence of tricyclic antidepressant and low SHAP values for PTB were positioned in the left bottom, whereas points with high TMD values, the presence of tricyclic antidepressant and high SHAP values for PTB were positioned in the right top.

### Contributions

The results of previous studies based on self-reported questionnaires [14–17] has been mixed on an association between PTB and TMD. The relationship was not statistically significant between control and experimental adolescents in some examinations [14, 15], but positive in another investigation [16]. Indeed, a systematic review confirmed the former finding (no significance) [17]. The unique contribution of this study is that it used machine learning and population data for confirming the positive association between PTB and TMD. One plausible pathway between PTB and TMD would be vitamin deficiency as in the case of PTB-oral hygiene status [18]. Moreover, the findings of explainable artificial intelligence (SHAP) in this study sheds new light on an association among PTB, TMD and antidepressant medication (depression). A systematic review and a prospective cohort study reported that pregnant women experience intense stress and this becomes a significant risk factor for their PTB [19, 20]. Pregnant women would take antidepressants to relieve their mental burden but this is expected to cause a vicious cycle of furthering the risk of PTB: Three systematic reviews highlighted a positive relationship between antidepressant medication and PTB [21–23]. As addressed above, in a similar context, a prospective study of female students before the university entrance reported a positive association between stress and TMD [7]. This positive association between stress and TMD was affirmed by a review of 33 original studies [8] and a survey of 112 participants in a general hospital [9]. This study extends the horizon of existing literature by employing explainable artificial intelligence and population data for identifying an association among PTB, TMD, GERD and antidepressant medication (depression) together. Previous studies highlight behavioral, infectious, neuroendocrine and neuroinflammatory mechanisms between stress and PTB [24]. Likewise, one possible pathway between stress and TMD would be the hypothalamic–pituitary–adrenal axis, the serotoninergic and opioid systems [25]. These statements can be extended to include GERD. No examination has been done and more investigation is needed in this direction.

### Limitations

This study had some limitations. Firstly, this study did not examine etiological differences between preterm birth due to maternal and fetal indication, and preterm birth due to spontaneous preterm labor. Secondly, this study did not investigate possible mediating effects among independent variables. Thirdly, this study did not use recurrent neural networks (deep learning models) for the limited capacity of the computer server in the Korea National Health Insurance Service data analysis center during the study period. Fourthly, this study did not cover parameter tuning, using the default hyper-parameters of the random forest, i.e., the number of trees 100, the splitting criterion of GINI, the max depth of trees undetermined.

### Conclusions

A cutting-edge approach of explainable artificial intelligence highlights the strong associations of preterm birth with temporomandibular disorder, gastrointestinal diseases and antidepressant medication. Close surveillance is needed for pregnant women regarding these multiple risks at the same time.

## Supporting information

S1 Table Abbreviations. (DOC)

S2 Table ICD-10 code for preterm birth, temporomandibular disorder and gastrointestinal diseases. (DOC)

S3 Table ATC code for medication. (DOC)
