# Supplementary material for: Machine learning analysis with population data for the associations of preterm birth with temporomandibular disorder and gastrointestinal diseases
Source: PLoS One. 2024 Jan 2;19(1):e0296329. doi: 10.1371/journal.pone.0296329 (PMC10760735; doi:10.1371/journal.pone.0296329)
Supplement: S1 Table — (DOC) [file pone.0296329.s001.doc]

**Table S1. Abbreviations**

| **Abbreviation** |  | **Description** |
| --- | --- | --- |
| AUC |  | Area Under the Receiver-Operating-Characteristic Curve |
| GERD |  | Gastroesophageal Reflux Disease |
| IRB |  | Institutional Review Board |
| PROM |  | Premature Rupture of Membranes |
| PTB |  | Preterm Birth |
| SHAP |  | Shapley Additive Explanation |
| TMD |  | Temporomandibular Disorder |
