# Supplementary material for: Machine learning analysis with population data for the associations of preterm birth with temporomandibular disorder and gastrointestinal diseases
Source: PLoS One. 2024 Jan 2;19(1):e0296329. doi: 10.1371/journal.pone.0296329 (PMC10760735; doi:10.1371/journal.pone.0296329)
Supplement: S2 Table — (DOC) [file pone.0296329.s002.doc]

**Table S2. ICD-10 Code for Preterm Birth, Temporomandibular Disorder and Gastrointestinal diseases**

|  |  | **Code** | **Description** |
| --- | --- | --- | --- |
| PTB | PROM | O42.00  O42.01  O42.10  O42.11  O42.20  O42.21  O42.90  O42.91 | Onset of labor within 24 hours of rupture (0-33 weeks of gestation)  Onset of labor within 24 hours of rupture (34-36 weeks of gestation)  Onset of labor after 24 hours of rupture (0-33 weeks of gestation)  Onset of labor after 24 hours of rupture (34-36 weeks of gestation)  Prolonged labor because of treatment (0-33 weeks of gestation)  Prolonged labor because of treatment (34-36 weeks of gestation)  Unspecified length of time between rupture and onset of labor (0-33 weeks of gestation)  Unspecified length of time between rupture and onset of labor (34-36 weeks of gestation) |
|  | PTL | O60.11  O60.12  O60.19 | Preterm delivery with preterm labor (0-33 weeks of gestation)  Preterm delivery with preterm labor (34-36 weeks of gestation)  Preterm delivery with preterm labor (unspecified weeks of gestation) |
|  | Other | O60.31  O60.32  O60.39 | Preterm delivery without preterm labor (0-33 weeks of gestation)  Preterm delivery without preterm labor (34-36 weeks of gestation)  Preterm delivery without preterm labor (unspecified weeks of gestation) |
| Dental Cavity |  | K02.0  K02.1  K02.2  K02.3  K02.5  K02.8  K02.9 | Caries limited to enamel  Caries of dentine  Caries of cementum  Arrested dental caries  Caries with pulp exposure  Other dental caries  Dental caries, unspecified |
| Periodontitis |  | K052  K0528  K0529  K053  K0538  K0539 | Aggressive periodontitis  Other aggressive periodontitis  Unspecified aggressive periodontitis  Chronic periodontitis  Other chronic periodontitis  Unspecified chronic periodontitis |
| Salivary Gland Disease |  | K11 | Salivary gland disease |
| Tooth Loss |  | K081 | Loss of teeth due to accident, extraction or local periodontal disease |
| TMD |  | K07.6 | Temporomandibular disorder |
| Crohn’s Disease |  | K50 | Crohn’s disease |
| GERD |  | K210 | Gastroesophageal reflux disease with esophagitis |
| Irritable Bowel Syndrome |  | K58.1  K58.2  K58.3  K58.8 | [Irritable bowel syndrome](http://wikipedia.org/wiki/Irritable_bowel_syndrome) with predominant [diarrhoea](http://wikipedia.org/wiki/Diarrhoea) [IBS-D]  [Irritable bowel syndrome](http://wikipedia.org/wiki/Irritable_bowel_syndrome) with predominant [constipation](http://wikipedia.org/wiki/Constipation) [IBS-C]  [Irritable bowel syndrome](http://wikipedia.org/wiki/Irritable_bowel_syndrome) with mixed bowel habits [IBS-M]  Other and unspecified [irritable bowel syndrome](http://wikipedia.org/wiki/Irritable_bowel_syndrome) |
| Ulcerative Colitis |  | K51 | Ulcerative colitis |
| Infertility |  | N96  N97  N98 | [Habitual aborter](http://wikipedia.org/wiki/Habitual_aborter)  [Female infertility](http://wikipedia.org/wiki/Female_infertility)  [Complications](http://wikipedia.org/wiki/Complication_(medicine)) associated with [artificial fertilization](http://wikipedia.org/wiki/Artificial_fertilization) |
| Hypertension |  | O10  O10.0  O10.4  O10.9  O11  O13  O14  O16  I10  I10.1  I10.9  I15  I15.0  I15.1  I15.2  I15.22  I15.28  I15.8  I15.80  I15.88  I15.9  I270  I272 | Pre-existing [hypertension](http://wikipedia.org/wiki/Hypertension) complicating [pregnancy](http://wikipedia.org/wiki/Pregnancy), [childbirth](http://wikipedia.org/wiki/Childbirth) and the [puerperium](http://wikipedia.org/wiki/Puerperium)  Pre-existing essential [hypertension](http://wikipedia.org/wiki/Hypertension) complicating [pregnancy](http://wikipedia.org/wiki/Pregnancy), [childbirth](http://wikipedia.org/wiki/Childbirth) and the [puerperium](http://wikipedia.org/wiki/Puerperium)  Pre-existing [secondary hypertension](http://wikipedia.org/wiki/Secondary_hypertension) complicating [pregnancy](http://wikipedia.org/wiki/Pregnancy), [childbirth](http://wikipedia.org/wiki/Childbirth) and the [puerperium](http://wikipedia.org/wiki/Puerperium)  Unspecified pre-existing [hypertension](http://wikipedia.org/wiki/Hypertension) complicating [pregnancy](http://wikipedia.org/wiki/Pregnancy), [childbirth](http://wikipedia.org/wiki/Childbirth) and the [puerperium](http://wikipedia.org/wiki/Puerperium)  Pre-existing hypertension with pre-eclampsia  Gestational [pregnancy-induced] hypertension without significant proteinuria  [Pre-eclampsia](http://wikipedia.org/wiki/Pre-eclampsia) superimposed on [chronic](http://wikipedia.org/wiki/Chronic_(medicine)) [hypertension](http://wikipedia.org/wiki/Hypertension)  [Gestational](http://wikipedia.org/wiki/Gestational) [pregnancy-induced] [hypertension](http://wikipedia.org/wiki/Hypertension)  [Pre-eclampsia](http://wikipedia.org/wiki/Pre-eclampsia)  Unspecified [maternal](http://wikipedia.org/wiki/Maternal) [hypertension](http://wikipedia.org/wiki/Hypertension)  Essential (primary) [hypertension](http://wikipedia.org/wiki/Hypertension)  Malignant hypertension  Other and unspecified primary [hypertension](http://wikipedia.org/wiki/Hypertension)  [Secondary hypertension](http://wikipedia.org/wiki/Secondary_hypertension)  [Renovascular hypertension](http://wikipedia.org/wiki/Renovascular_hypertension)  [Hypertension](http://wikipedia.org/wiki/Hypertension) secondary to other [renal](http://wikipedia.org/wiki/Renal) disorders  [Hypertension](http://wikipedia.org/wiki/Hypertension) secondary to [endocrine disorders](http://wikipedia.org/wiki/Endocrine_disorders)  Other [secondary hypertension](http://wikipedia.org/wiki/Secondary_hypertension)  Hypertension secondary to drug  Other [secondary hypertension](http://wikipedia.org/wiki/Secondary_hypertension)  [Secondary hypertension](http://wikipedia.org/wiki/Secondary_hypertension), unspecified  [Primary pulmonary hypertension](http://wikipedia.org/wiki/Primary_pulmonary_hypertension)  Other [secondary pulmonary hypertension](http://wikipedia.org/wiki/Secondary_pulmonary_hypertension) |
| Diabetes |  | E10  E11  E12  E13  E14  O24.0  O24.1  O24.2  O24.3 | Type 1 [diabetes mellitus](http://wikipedia.org/wiki/Diabetes_mellitus)  Type 2 [diabetes mellitus](http://wikipedia.org/wiki/Diabetes_mellitus)  Malnutrition-related [diabetes mellitus](http://wikipedia.org/wiki/Diabetes_mellitus)  Other specified [diabetes mellitus](http://wikipedia.org/wiki/Diabetes_mellitus)  Unspecified [diabetes mellitus](http://wikipedia.org/wiki/Diabetes_mellitus)  Pre-existing type 1 [diabetes mellitus](http://wikipedia.org/wiki/Diabetes_mellitus)  Pre-existing type 2 [diabetes mellitus](http://wikipedia.org/wiki/Diabetes_mellitus)  Pre-existing malnutrition-related [diabetes mellitus](http://wikipedia.org/wiki/Diabetes_mellitus)  Pre-existing [diabetes mellitus](http://wikipedia.org/wiki/Diabetes_mellitus), unspecified |
| Gestational Diabetes |  | O24.4  O24.9 | [Diabetes mellitus](http://wikipedia.org/wiki/Diabetes_mellitus) arising in [pregnancy](http://wikipedia.org/wiki/Pregnancy)  [Diabetes mellitus](http://wikipedia.org/wiki/Diabetes_mellitus) in [pregnancy](http://wikipedia.org/wiki/Pregnancy), unspecified |

Abbreviations: PTB Preterm Birth, PROM Premature Rupture of Membranes, PTL Preterm Labor with Preterm Delivery, TMD Temporomandibular Disorder, GERD gastroesophageal reflux disease
