# Supplementary material for: Machine learning analysis with population data for the associations of preterm birth with temporomandibular disorder and gastrointestinal diseases
Source: PLoS One. 2024 Jan 2;19(1):e0296329. doi: 10.1371/journal.pone.0296329 (PMC10760735; doi:10.1371/journal.pone.0296329)
Supplement: S3 Table — (DOC) [file pone.0296329.s003.doc]

**Table S3. ATC Code for Medication**

| **Medication** | **Code** | **Description** | |
| --- | --- | --- | --- |
| Benzodiazepine | N05BA | Benzodiazepine derivatives | (N05 Psychoanaleptics, N05B Anxiolytics) |
|  | N05CD | Benzodiazepine derivatives | (N05 Psychoanaleptics, N05C Hypnotics and sedatives) |
|  | N05CF | Benzodiazepine-related drugs | (N05 Psychoanaleptics, N05C Hypnotics and sedatives) |
| Calcium Channel Blocker | C08 | Calcium channel blockers | |
| Nitrate | C01DA | Organic nitrates | |
| Progesterone | G03 | Sex hormones and modulators of the genital system | |
| Tricyclic Antidepressant | N06A | Tricyclic antidepressants | |
| Sleeping Pills | N05C | Hypnotics and sedatives | |
